# Nutrient deprivation in neuroblastoma cells alters 4-hydroxynonenal-induced stress response

**DOI:** 10.18632/oncotarget.14132

**Published:** 2016-12-24

**Authors:** Lars Zimmermann, Rudolf Moldzio, Katarina Vazdar, Christopher Krewenka, Elena E. Pohl

**Affiliations:** ^1^ Institute of Physiology, Pathophysiology and Biophysics, Department of Biomedical Sciences, University of Veterinary Medicine, Vienna, Austria; ^2^ Institute of Medical Biochemistry, Department of Biomedical Sciences, University of Veterinary Medicine, Vienna, Austria; ^3^ Division of Organic Chemistry and Biochemistry, Rudjer Boskovic Institute, Zagreb, Croatia

**Keywords:** cancer cell metabolism, mitochondrial membrane potential, starvation, oxononenal, ß-oxidation

## Abstract

4-hydroxy-2-nonenal (HNE), a toxic lipid peroxidation product, is associated with oxidative damage in cells and involved in various diseases including the initiation and progression of cancer. Cancer cells have a high, adaptable metabolism with a shift from oxidative phosphorylation to glycolysis and rely on high levels of glucose and glutamine as essential nutrients for cell growth. Here we investigated whether the toxic effects of HNE on the mitochondrial membrane potential (MMP) of cancer cells depends on their metabolic state by deprivation of glucose and/or glutamine. The addition of 16 μM HNE to N18TG2 neuroblastoma cells incubated in glucose medium led to a severe reduction of MMP, which was similar to the MMP of cells fed with both glucose and glutamine. In contrast, HNE addition to cells starved in glutamine medium increased their MMP slightly for a prolonged time period and this was accompanied by increased cellular survival. We found that ß-oxidation of HNE did not cause the increased MMP, since the aldehyde dehydrogenase was distinctly more active in cells with glucose medium. However, after blocking fatty acid ß-oxidation in cells starved in glutamine medium with etomoxir, which inhibits carnitine palmitoyltransferase 1, HNE addition induced a strong reduction of MMP similar to cells in glucose medium. Surprisingly, the effect of more toxic 4-oxo-2-nonenal was less pronounced. Our results suggest that in contrast to cells fed with glucose, glutamine-fed cancer cells are capable of ß-oxidizing fatty acids to maintain their MMP to combat the toxic effects of HNE.

## INTRODUCTION

Mitochondria are the major source for the generation of ROS such as superoxide, hydrogen peroxide and lipid hydroperoxide products [1–3]. Reactive aldehydes such as HNE, oxononenal (ONE) and others are generally formed by oxidation of ω-6 polyunsaturated fatty acids (PUFAs) such as arachidonic acid or linoleic acid by proton abstracting agents (e.g. hydroxyl radicals) [4, 5]. HNE may also be generated by oxidation of cardiolipin [6], which is a component of the inner mitochondrial membrane. HNE interacts with DNA, proteins containing the amino acids cysteine, lysine or histidine, and lipids containing amino groups (e.g. phosphatidylethanolamine) [7–10]. With proteins and lipids HNE forms Michael and Schiff base adducts [11, 12].

The physiological level of HNE in plasma is in the range of 0.07-0.7 μM [13, 14]. At these concentrations HNE was reported to be involved in cellular signalling pathways to regulate proliferation, differentiation and apoptosis [14–16] and to control the cellular antioxidant defence system [17–19]. Under toxic conditions the local concentrations can be quite high and reach 100 μM at the plasma membrane [20] leading to oxidative damage, loss of mitochondrial membrane potential (MMP) and cell death.

HNE is degraded rapidly within cells, in hepatocytes the half-life of 100 μM HNE is ~5 min [21], in human endothelial cells 95% of 5 μM HNE is removed within 30 minutes [22]. There are three major pathways for HNE detoxification. (i) The majority of HNE is removed by reacting with abundant antioxidant glutathione directly or via glutathione-S-transferases, (ii) alcohol dehydrogenases or aldo-keto reductases convert HNE to 1,4-dihydroxy-2-nonene (DHN); and iii) aldehyde dehydrogenases (ALDHs) oxidize HNE to 4-hydroxy-2-nonenoic acid (HNA) [23]. HNE adducts can be removed from the cell by excretion or by autophagic and proteosomal degradation [24, 25].

HNE affects various cellular pathways, which change cell proliferation and survival, mitochondrial function and cellular metabolism [26] and has thus been associated with the initiation and progression of cancer [27, 28]. Cancer cells in general have a high cellular metabolism with a higher glucose uptake and a shift from oxidative phosphorylation to aerobic glycolysis, the so-called Warburg effect [29]. This higher metabolism typically leads to higher production of HNE, which is counteracted by an increased antioxidant system in cancer cells with elevated levels of ALDHs, superoxide dismutases, thioredoxins, glutaredoxins and peroxiredoxins [30–33]. Additionally, the enhanced glucose metabolism stimulates the production of pyruvate and NADPH, that helps to detoxify HNE by increasing levels of the reduced form of glutathione [34]. Furthermore, cancer cells increase the conversion of glutamine, which is essential for cancer growth, into glutathione to reduce HNE levels [35].

Since cancer cell growth and survival strongly depend on high levels of glucose and glutamine as nutrients, deprivation of these nutrients has been utilized as anti-cancer treatment, although this application has been hampered, since cancer cells have the ability to adapt their metabolism [35, 36]. Glucose deprivation in cancer cells is associated with inhibition of HNE detoxification due to reduced levels of NADPH and pyruvate leading to increased oxidative stress and potentially cell death [37, 38]. Inhibition of cancer cell growth has also been shown by deprivation of glutamine [39]. Both glucose and glutamine deprivation increase autophagy [40–42], which in succession promotes cellular survival by recycling cellular compartments and by protecting against oxidative damage. However, it is still not understood how HNE, generated by oxidative damage, acts on the mitochondrial metabolism under such deprivation conditions, and whether there is a difference between glucose and glutamine deprivation. In differentiated SH-SY5Y neuroblastoma cells, deprivation of glucose increased HNE-induced toxicity [38]. However, cells were deprived of glucose by inhibition of glycolysis with either 2-deoxyglucose or koningic acid, which both also inhibited the autophagic flux. The latter could be an explanation for higher HNE toxicity.

In this work, we aimed to investigate whether the toxic effect of HNE on the MMP of cancer cells depends on metabolic conditions. For this, murine N18TG2 neuroblastoma cells were incubated in medium containing either glucose or glutamine as a nutrient or both glucose and glutamine before HNE was added and then changes of the MMP and cell viability were measured. To gain mechanistic insight, we used inhibitors against involved metabolic pathways. Additionally, we analysed whether our observed effects are specific for HNE by measuring the more reactive aldehyde ONE.

## RESULTS AND DISCUSSION

### Addition of 4-hydroxy-2-nonenal (HNE) to N18TG2 neuroblastoma cells decreases mitochondrial membrane potential (MMP) and changes cellular morphology

**Figure 1:**
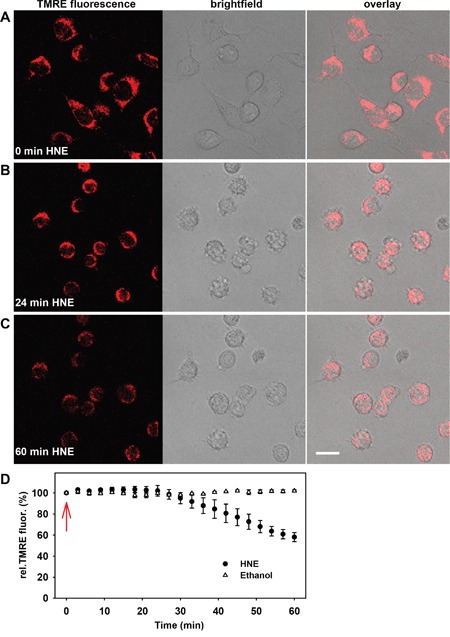
Alteration of the mitochondrial membrane potential and cellular morphology after addition of 4-hydroxy- 2-nonenal (HNE) to N18TG2 neuroblastoma cells in a typical experiment **A-C.** Fluorescence (left), brightfield (middle) and merged (right) images of the cells were recorded **A.** without HNE, **B.** 24 minutes and **C.** 60 minutes after addition of 16 μM HNE. Cell medium contained 21.6 mM glucose, 3.85 mM glutamine, 9.6% fetal bovine serum and 1.92 mM sodium pyruvate. Scale bar is 20 μm. **D.** Time course of normalized TMRE fluorescence after addition of 16 μM HNE and ethanol as a control (arrow). TMRE fluorescence was normalized to the fluorescence at time point 0 min. Data are presented as mean values ± SEM from at least 5 independent experiments.

As a cancer cell model, we used murine N18TG2 neuroblastoma cells, which have high levels of acetylcholinesterase and are unable to establish synaptic contacts, because the choline acetyltransferase is hardly expressed. First, we tested the effect of HNE on MMP under normal nutrient conditions (DMEM medium with 21.6 mM glucose, 3.85 mM glutamine, 9.6% fetal bovine serum and 1.92 sodium pyruvate) at 37°C and 5% CO_2_. To track MMP changes, cells at a cell density of 100 cells/mm^2^ were incubated for 20 min with the potential sensitive dye tetramethylrhodamine (TMRE, 12.5 nM) (Figure 1A). Then HNE was dissolved in ethanol and added to cells at a final concentration of 16 μM. Fluorescence change was determined every 3 minutes for one hour by recording z-stacks using a confocal laser scanning microscope (Figure 1B–1D). Since 16 μM HNE is already rather toxic for cells [43], its addition led to a strong reduction of MMP by about 45% within one hour of incubation (Figure 1C). Control measurements with ethanol had no effect on the MMP (Figure 1D). Alongside with the effects on the MMP, HNE also induced changes in cells morphology even after 5-10 minutes after its addition (Figure 1B).

**Figure 2:**
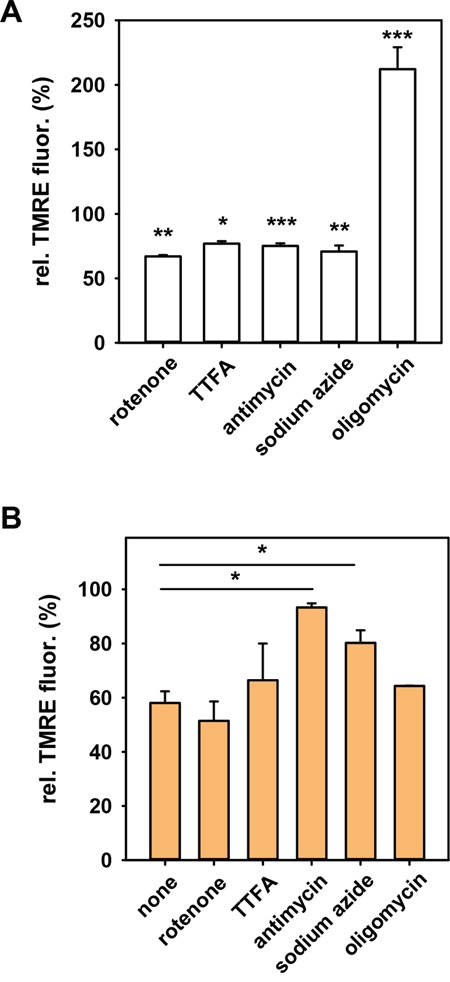
Effect of respiratory chain complexes inhibition on the mitochondrial membrane potential in N18TG2 cells **A.** in the absence and **B.** in the presence of HNE. TMRE-stained cells were incubated with inhibitors of complex I (10 nM rotenone), complex II (0.5 mM TTFA), complex III (50 μM antimycin A), complex IV (10 μM sodium azide) and complex V (2 μM oligomycin). TMRE fluorescence measured 30 minutes after addition of inhibitors was normalized to the fluorescence in the absence of the inhibitors (control with ethanol). The final concentration of HNE was 16 μM. Data are presented as mean values ± SEM. * p < 0.05, ** p < 0.01, *** p < 0.001.

Data for several cancer cell lines has shown that mitochondria are dysfunctional in regard to energy production and the MMP is hyperpolarized [44–46], but other cancer cell lines exhibit active oxidative phosphorylation [47, 48]. We found that N18TG2 cells possess functional, energy-producing mitochondria, which can be seen, above all, by the strong increase of MMP after addition of oligomycin (Figure 2A). Next, we investigated whether modification of respiratory chain complexes by HNE causes the observed reduction of MMP. For this, we inhibited each complex for 30 min with an appropriate inhibitor (Figure 2A) and subsequently determined whether HNE-induced MMP reduction was affected. In the presence of complex III inhibitor antimycin or complex IV inhibitor sodium azide, HNE just slightly reduced the MMP (Figure 2B), indicating that the activity of these two complexes are affected the most by HNE modification, which is in line with other studies [49]. In contrast, inhibition of complexes I, II and V (ATP-synthase) with rotenone, thenoyltrifluoroacetone or oligomycin hardly affected the HNE-mediated MMP decrease.

The effect of HNE on the MMP was dependent on the cell concentration. For example, for a sixfold higher cell concentration (600 cells/mm^2^) 64 μM HNE was necessary for the same MMP alteration as 16 μM at the lower cell density. Therefore, we kept the cell density for each measurement constant at 100 cells/mm^2^.

### HNE-mediated MMP alteration depends on the type of available nutrient

**Figure 3:**
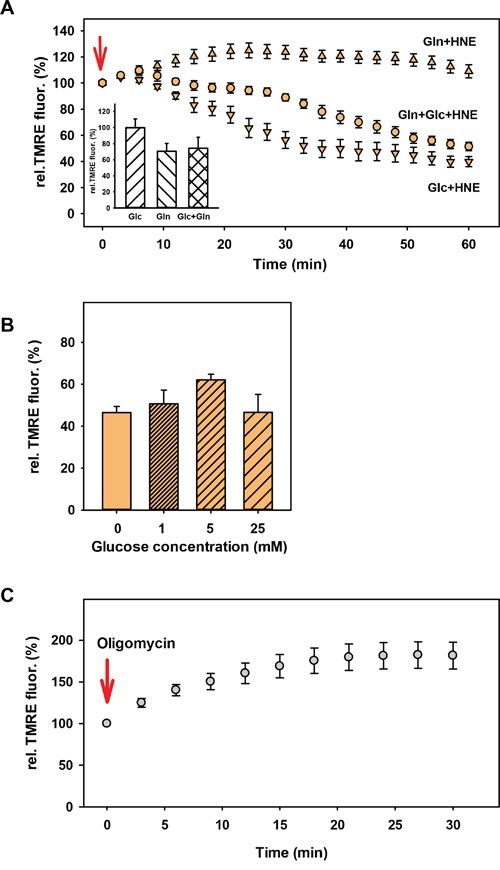
Dependence of the HNE-induced mitochondrial membrane potential alteration of N18TG2 cells on nutrient supply **A.** DMEM medium contained 25 mM glucose (down-pointing triangles; Glc), 3.85 mM glutamine (up-pointing triangles; Gln) or 24.5 mM glucose + 3.85 mM glutamine (circles; Gln+Glc). TMRE fluorescence in the presence of HNE was normalized to the TMRE fluorescence without HNE (insert). The concentration of HNE (arrow indicates the addition) was 16 μM. Data are presented as mean values ± SEM for 5-10 independent experiments. **B.** TMRE-stained cells were incubated for 2 h 30 min in DMEM medium with different glucose concentrations before addition of 16 μM HNE. TMRE fluorescence after 1h HNE treatment normalized to the fluorescence before HNE addition is shown. Data are presented as mean values ± SEM for 3 independent experiments. **C.** TMRE stained cells were starved for 2 h 30 min in 3.85 mM glutamine medium. TMRE fluorescence after addition of 2 μM oligomycin was normalized to the fluorescence without oligomycin. Data are presented as mean values ± SEM for 4 independent experiments.

Depending on the medium, cancer cells can adapt their metabolism under starvation conditions either in the direction of glycolysis in the presence of only glucose or oxidative phosphorylation, if only glutamine is present [50]. Next, we determined whether the action of HNE on the MMP is affected by the type of nutrient deprivation. As a control, we analysed the effect of HNE on cells incubated in DMEM medium containing both 24.5 mM glucose and 3.85 mM glutamine, but no FCS and pyruvate. Incubation for 2 h 30 min in this medium without HNE reduced the MMP by ~25% (Figure 3A; insert). The subsequent addition of HNE led to a strong reduction of the MMP (50%) similar to that which was observed in the full medium (45%, Figure 1D). We revealed that the 2h 30 min starvation in DMEM medium containing 25 mM glucose, but no glutamine (from now on referred as “glucose medium”), had no effect on the MMP (Figure 3A; insert). HNE addition led to an even stronger reduction of the MMP (Figure 3A, down-pointing triangles) compared to the experiment with cells incubated with both glucose and glutamine (Figure 3A, circles). Cell starvation in DMEM medium containing 3.85 mM glutamine but no glucose (from now on referred as “glutamine medium”) reduced the MMP by ~30% (Figure 3A; insert). Interestingly, the addition of HNE to these cells did not decrease the MMP within the same incubation time (Figure 3A, up-pointing triangles). It was even slightly increased by ~20% during the first hour, probably due to higher substrate availability for oxidative phosphorylation.

We also tested whether the strong difference in the HNE-altered MMP between cells kept in glucose and glutamine media is dependent on the glucose concentration, since the normal glucose concentration of the N18TG2 neuroblastoma medium is quite high (>20 mM). However, no difference between 1 mM, 5 mM and 25 mM glucose in the medium could be observed (Figure 3B). Also, DMEM alone, without any addition of further nutrients showed roughly the same reduction of the MMP after cell treatment with HNE as in the glucose medium (Figure 3B), indicating that both lack of glucose and the presence of glutamine are necessary for the maintenance of MMP.

### β-oxidation of HNE is not the reason for cells fed with glutamine to maintain MMP

To gain some mechanistic insight we first tested, whether the ATP synthase runs in reverse mode (ATP hydrolysis) under glucose deprivation keeping a high MMP, as described for several cell types under starvation conditions [51, 52]. For this, we added 2 μM oligomycin, the inhibitor of the ATP synthase, which was expected to reduce MMP in case the enzyme would work in reverse mode. However, Figure 3C shows that oligomycin addition to cells incubated 2 h 30 min in glutamine medium increased the MMP by ~80%, which indicates that the ATP synthase functioned in normal mode.

**Figure 4:**
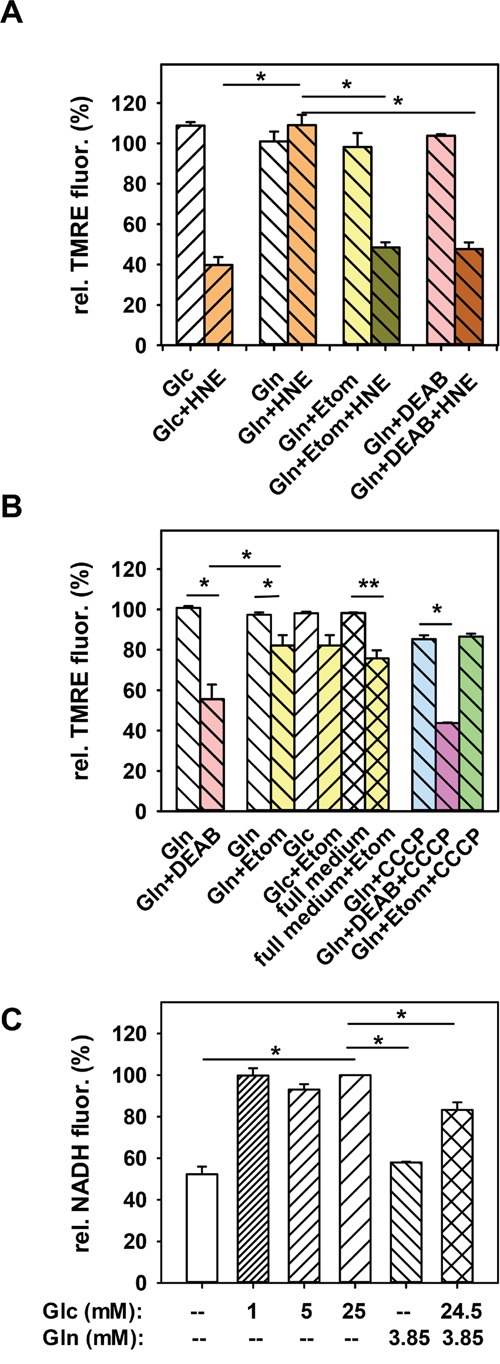
Dependence of the HNE-induced mitochondrial membrane potential alteration of N18TG2 cells on β-oxidation **A.** In cells, incubated in 3.85 mM glutamine medium (Gln), either β-oxidation was inhibited with 100 μM etomoxir (Etom), or ALDH activity was blocked with 50 μM DEAB. Then 16 μM HNE was added for 1h. TMRE fluorescence after 1h incubation with HNE was normalized to the fluorescence before HNE addition. For comparison controls without inhibitors are shown (25 mM glucose medium (Glc) or Gln). Data are presented as mean values ± SEM for 3-10 independent experiments. **B.** Effect of etomoxir (Gln+Etom), DEAB (Gln+DEAB) and 1 μM CCCP on the MMP of cells in glutamine medium without HNE. Etomoxir was also added to full medium (full medium+Etom) and to cells incubated in glucose medium (Glc+Etom). White bars are controls without inhibitor. Data are presented as mean values ± SEM for 3-12 independent experiments. **C.** NADH levels of cells incubated in medium containing different glucose (Glc) or glutamine (Gln) concentrations. NADH fluorescence was normalized to NADH fluorescence of cells incubated 2 h 30 min in 25 mM glucose medium. Data are presented as mean values ± SEM for 200 cells from 2 independent experiments. *p < 0.05, **p < 0.01.

**Figure 5:**
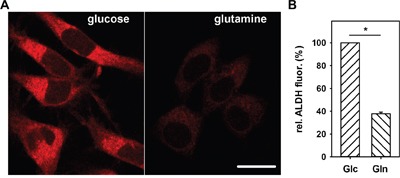
Nutrient dependence of AldeRed fluorescence in N18TG2 cells **A.** Typical fluorescence images of cells stained for 30 min with AldeRed after 2 h 30 min incubation in 25 mM glucose (left) or 3.85 mM glutamine (right) medium. Scale bar is 20 μm. **B.** Comparison of the relative AldeRed fluorescence changes in cells incubated in glucose (Glc) or glutamine (Gln) medium. Measured ALDH fluorescence was normalized to ALDH fluorescence of cells incubated in glucose medium. Data are presented as mean values ± SEM for 2 independent experiments, (>600 cells analyzed). * p < 0.05.

To test whether HNE is converted to 4-hydroxynonanoic acid (HNA) by the aldehyde dehydrogenase (ALDH), followed by its β-oxidation and subsequent increase of substrates for oxidative respiration, we blocked the ALDH by the competitive inhibitor diethylaminobenzaldehyde (DEAB, 50 μM) in cells kept in glutamine medium. Figure 4A shows that after pre-incubation with DEAB and after addition of HNE, cells could no longer maintain their MMP and its decrease was comparable to that observed in cells kept in glucose medium. Thus, we first hypothesized that β-oxidation of HNE is a likely cause for the slightly increased MMP in the presence of HNE. However, DEAB without HNE already had a strong effect on MMP, reducing it by ~44% (Figure 4B). Surprisingly, pre-incubation with DEAB also strongly increased the uncoupling effect of the artificial uncoupler CCCP in cells fed with glutamine (Figure 4B). Since CCCP is very unlikely related to the activity of the ALDH, it is possible that DEAB not only affects the activity of the ALDH, but also the stability of the mitochondrial membrane, which questions previous experiments with DEAB. Because it was difficult to conclude from these results, to what extent β-oxidation of HNE plays a role in maintaining MMP, we tested which difference between cells in glucose and glutamine medium could lead to a different capacity to β-oxidize HNE. Since the ALDH needs NAD^+^ for the oxidation of HNE to HNA, we suggested that different NAD^+^ levels between cells in glucose and glutamine media lead to different ALDH activity and thus different β-oxidation of HNE. Indeed, two-photon imaging revealed, that NADH fluorescence is roughly twice as high after 2 h 30 min starvation in 25 mM glucose medium compared to glutamine medium (Figure 4C), which corresponds to low NAD^+^ levels and thus potentially low ALDH activity. NADH levels in medium containing both glucose and glutamine were slightly lower than in medium containing only glucose. NADH levels in cells kept in 1 mM or 5 mM glucose were similar to those in cells kept in 25 mM glucose (Figure 4C), which would be in line with the MMP results after HNE treatment (Figure 3B). However, cells incubated in DMEM medium without any additional nutrients, which were not able to maintain their MMP after HNE treatment (Figure 3B), had similar low NADH levels as cells in glutamine medium (Figure 4C). Thus, a correlation between low NADH levels and the ability to maintain the MMP is hardly probable. Next, we directly tested the ALDH activity with the AldeRed ALDH detection assay [53]. Interestingly, the fluorescence and thus ALDH activity was significantly higher in cells with glucose medium compared to cells with glutamine medium (Figure 5), despite the lower NAD^+^ levels. Thus it is unlikely, that cells in glutamine medium exhibit a higher β-oxidation of HNE to maintain MMP.

### Cells fed with glutamine sustain MMP after addition of HNE via fatty acid β-oxidation

As a next step, we tested whether β-oxidation of fatty acids is the cause for the maintained MMP, since β-oxidation is typically up-regulated in starved cells without glucose [54]. To test this, we blocked β-oxidation in cells with glutamine medium with etomoxir before HNE treatment. It has been shown that incubation with etomoxir, which is an irreversible inhibitor of carnitine palmitoyltransferase-1 in the outer mitochondrial membrane, reduces production of ATP due to inhibition of β-oxidation [55]. It is important to mention, that the carnitine palmitoyltransferase system transports long chain fatty acids (LCFA, C12-C20), while fatty acids with shorter chains can diffuse passively over the mitochondrial membrane and get converted by the medium chain acyl-CoA dehydrogenase for subsequent β-oxidation. Thus, etomoxir should not influence β-oxidation of HNA (C9). To exclude the additional toxicity of etomoxir which reduces GSH and NADPH levels at high concentrations (1 mM, [56, 57]), we used a relatively low concentration of 100 μM, previously shown to be safe in human glioblastoma SF188 cells [56]. Addition of 100 μM etomoxir to cells in glutamine medium without HNE reduced MMP by ~20% after 30 minutes, similar to results with cells in glucose or full medium (Figure 4C). This indicates that at this time point, β-oxidation of fatty acids is not significantly up-regulated in cells incubated in glutamine medium. In this regard, it was now surprising that cells treated with etomoxir in glutamine medium could no longer maintain their MMP after addition of HNE, and the decrease in MMP was similar to that observed in experiments with cells in glucose medium (Figure 4B). It means that cells fed with glutamine without glucose increased the β-oxidation of fatty acids only during an increased stress situation such as the rise of HNE levels. We tested, whether pre-incubation with etomoxir would alter CCCP uncoupling in cells fed with glutamine, which was not the case (Figure 4C). Thus, we conclude that β-oxidation of fatty acids, but not of HNE, is the likely cause for the maintained MMP after HNE treatment in cells incubated in glutamine medium.

### Cell viability after HNE treatment differs between glutamine- and glucose-fed cells

**Figure 6:**
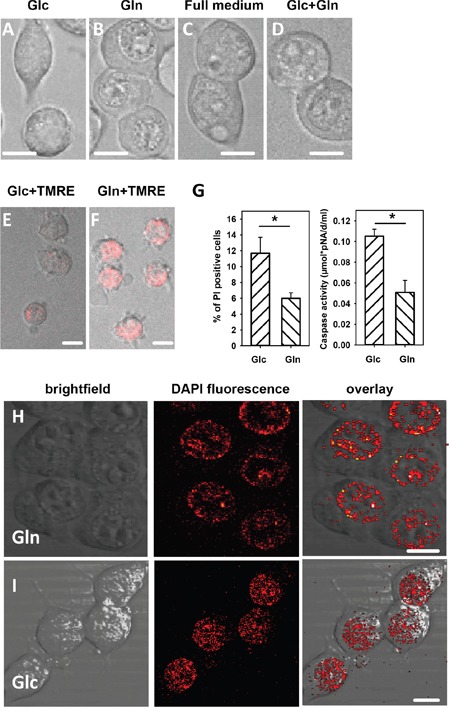
Morphology, viability and nucleus morphology of cells incubated in different media after addition of HNE observed with brightfield and fluorescent microscopy Cells were incubated for 150 min in medium containing either **A., E.** and **I.** 25 mM glucose, or **B., F.** and **H.** 3.85 mM glutamine medium, or **C.** full medium, or **D.** 24.5 mM glucose and 3.85 mM glutamine. The concentration of HNE added for 1 h was 16 μM. **A-D.** Morphology, **E** and **F.** Reversibility of the mitochondrial membrane potential: cells were washed after HNE treatment and incubated for 3 hours in fresh medium with TMRE. **G.** Viability: The percentage of propidium iodide (PI) positive cells and caspase 3 activity was determined after 1 h incubation with HNE. Data are presented as mean values ± SEM for 5 independent experiments. * p < 0.05. **H** and **I.** Cell nuclei were stained with DAPI. Scale bar is 10 μm.

HNE treatment led to a strong stress response in N18TG2 cells (Figure 1). Interestingly, the morphology of the cells was very different after 1h HNE treatment between cells in glucose and glutamine medium, especially regarding the nucleus (Figure 6A–6B). In cells fed with glutamine as a main nutrient, the volume ratio between cytosol to nucleus remained unchanged after treatment with HNE. In cells kept in glucose medium, the nucleus filled the major portion of the cell volume, which was confirmed by DAPI staining (Figure 6I). The morphological change of HNE-treated cells in glucose medium cannot be attributed to the MMP decrease, since cells in full medium and in medium containing both glucose and glutamine, which exhibit a loss of MMP (Figure 1D; Figure 3A), had a morphology similar to cells kept in glutamine (Figure 6C–6D) after HNE treatment. This indicates that a lack of glutamine may be the cause for the morphological change. We further analysed cellular survival by staining cells with propidium iodide (PI). We found an increased percentage of dead cells after 1 h HNE treatment with glucose medium compared to glutamine medium (Figure 6G). Also the increased caspase 3 activity in HNE-treated cells (Figure 6G) confirmed the higher cell apoptosis in glucose medium compared to glutamine medium.

To evaluate whether the cells would recover from the severe HNE treatment, we washed the cells after 1 h HNE treatment, and incubated them for 3 h in fresh full medium, before determining MMP. Cells treated in glucose media could not recover their low MMP in contrast to the cells in glutamine whose MMP was roughly double as high as in the cells treated in glucose media after 3 h recovery (Figure 6E–6F). Even if observed one day after HNE treatment, cells treated in glutamine media still exhibited a distinct TMRE fluorescence, which could not be observed in glucose-fed cells.

It is generally accepted that HNE acts on cells differently dependent on its concentration (s. Introduction) and cell type. For example, it has been shown, that HNE induces apoptosis via inactivation of membrane-associated catalase in cancer cells but not in normal cells [58]. However, at lower concentrations, HNE can act as a cell signalling molecule to alter several cellular functions such as increasing cellular survival against oxidative stress or it can impact cell differentiation, cell cycle, cell growth or cellular metabolism. Our results show, that the toxicity of HNE is dependent on the metabolic state of cancer cells and HNE can also induce cellular signalling to enhance cell survival by increasing cellular β-oxidation even at high toxic HNE concentrations, but only if just glutamine is present as a nutrient.

Our results differ considerably from those observed previously in SH-SY5Y neuroblastoma cells [38]. Dodson et al. showed that after starvation, HNE induced an increased cell death and strongly reduced MMP, while the effects without starvation were very weak. This discrepancy could be explained by three main differences in the experimental system employed. (i) Most importantly, Dodson et al. deprived glucose by blocking glycolysis with 2-deoxyglucose or koningic acid, which inhibits the autophagic flux and thus drastically increases HNE toxicity; (ii) differentiated neuroblastoma cells were used in experiments, where metabolism was different compared to undifferentiated cells [59]; and iii) cells were starved for 24 hours compared to 2 h 30 min starvation applied in our experiments.

### 4-oxo-2-nonenal acts stronger than HNE, but the difference between glucose- and glutamine-fed cells is strongly diminished

**Figure 7:**
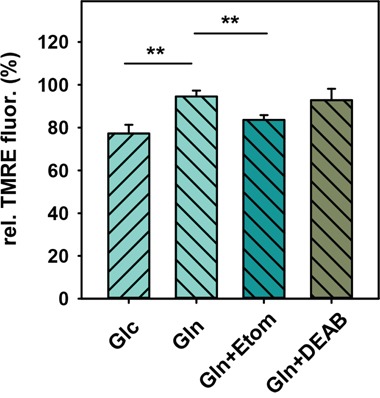
Effect of 4-oxo-2-nonenal (ONE) on alteration of mitochondrial membrane potential Cells were stained with TMRE and incubated for 2 h 30 min in 25 mM glucose medium (Glc), 3.85 mM glutamine medium (Gln), 50 μM DEAB + 3.85 mM glutamine medium (Gln+DEAB) or 100 μM etomoxir + 3.85 mM glutamine medium (Gln+Etom) before 0.75 μM ONE was added. DEAB or etomoxir were added to the cells 75 or 120 min after the start of starvation. The TMRE fluorescence after 1h incubation with ONE was normalized to the fluorescence before ONE addition. Data are presented as mean values ± SEM for 8-13 independent experiments. **p < 0.01.

Finally, we tested whether these results are specific for HNE or whether reactive aldehydes known to be more toxic behave similarly. For this we determined the effect of 4-oxo-2-nonenal (ONE) on the MMP under different and the same starvation conditions as for HNE. Indeed, we found that ~ 0.75 – 1 μM ONE had roughly the same effect on the MMP as 16 μM HNE in medium with both glucose and glutamine (Figure 7). Next, we tested the difference in MMP alteration between cells kept in glucose and glutamine media after ONE treatment. Figure 7 shows that while in glutamine medium, the cells could maintain their MMP for 1h in the presence of 0.75 μM ONE, the MMP in cells kept in glucose media were reduced by ~25%. Remarkably, this difference between both media was much smaller compared to HNE (25% against 65%). MMP never increased above 100% in glutamine-fed cells, which is also in contrast to HNE. Blocking β-oxidation with etomoxir in cells kept in glutamine medium led to a reduction of the MMP closer to the levels of cells with glucose, although not reaching the same levels. Pre-incubation with DEAB did not alter the effect of ONE on the MMP in cells incubated in glutamine medium in contrast to the results observed with HNE. An explanation for these results might be the differences in interaction of HNE and ONE with membranes by formation of various adducts, which may have a different impact on metabolic pathways [12].

## CONCLUSION

**Figure 8:**
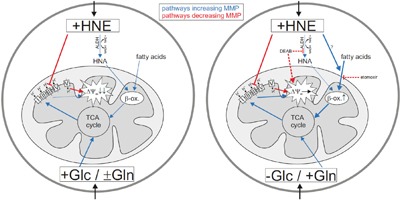
Mechanism of the HNE-induced alteration of mitochondrial membrane potential (MMP) in nutrient-deprived cancer cells Left: Cancer cells in glucose medium with or without glutamine. Right: Cancer cells in glutamine medium. In red are pathways leading to a reduction of MMP, in blue MMP increasing pathways. Thick lines indicate pathway activation. Dashed lines indicate the effects of the inhibitors DEAB and etomoxir.

In summary, our results show that HNE's effect on cancer cells depends on the type of nutrient deprivation used (Figure 8). Under conditions of glucose deprivation, where the cells still have glutamine, cancer cells can adapt to oxidative stress induced by incubation with HNE by increasing β-oxidation of fatty acids. This allows cells to maintain their MMP for a prolonged period in contrast to cells in glucose medium deprived of glutamine, where the MMP was severely reduced due to HNE addition. However, HNE strongly reduced MMP even in media, where both glucose and glutamine were present, indicating that in order to maintain the MMP via β-oxidation, cells have to be deprived of glucose and glutamine must be present. Cell viability results show that the cells in glutamine medium exhibited a markedly better cell survival against HNE toxicity compared to cells in glucose medium. Our work gives further mechanistic insights into oxidative stress in nutrient-deprived cancer cells, which could improve anti-cancer treatments based on nutrient deprivation.

## MATERIALS AND METHODS

### Chemicals

TMRE (tetramethyl rhodamine ethyl ester), DEAB (diethylaminobenzaldehyde), etomoxir, rotenone, antimycin A, TTFA (thenoyltrifluoroacetone), sodium azide and oligomycin were purchased from Sigma Aldrich. DAPI were purchased from Cell Signaling Technology^@^, propidium iodide (PI) from Invitrogen. HNE and ONE, dissolved in ethanol, were either obtained from Cayman Chemicals, or produced by ourselves (see below).

### Synthesis of (E)-4-hydroxy-2-nonenal (4-HNE)

1-Octen-3-ol (153 μL, 1.0 mM, 1.0 equiv) was dissolved in 5 mL of dry dichloromethane under argon atmosphere and the mixture degassed. Then acrolein (223 μL, 3.0 mM, 3.0 equiv) was added, followed by 1.25 mol% of Grubbs-Hoveyda 2^nd^ generation catalyst. The reaction was stirred at RT and after 3 h another 1.25 mol% of catalyst was added (total 16 mg, 0.025 mM, 0.025 equiv). The reaction mixture was further stirred for 15 h and evaporated. The crude product was purified by flash chromatography on silica using ethyl-acetate:hexane = 1:2 as eluent yielding 144 mg (92%) of product. All spectroscopic data for 4-HNE were in accordance with the previously reported [60] and were as follows for ^1^H NMR (CDCl_3_): δ 0.91 (t, *J* = 7.0 Hz, 3H), 1.26–1.44 (m, 6H), 1.61-1.69 (m, 2H), 1.83 (d, *J* = 4.5 Hz, 1H), 4.45 (m 1H), 6.32 (ddd, *J* = 1.5, 7.9 and 15.9 Hz, 1H), 6.83 (dd, *J* = 4.5, 15.9, 1H), 9.6 (d, *J* = 7.9 Hz, 1H).

### Synthesis of (E)-4-oxo-2-nonenal (4-ONE)

4-hydroxynonenal (19 mg, 0.12 mM, 1.0 equiv) was dissolved in 1 mL of dry dichloromethane under argon atmosphere. The mixture was cooled to 0 °C and the Dess-Martin oxidant (62 mg, 0.15 mM, 1.2 equiv) was added. The reaction mixture was further stirred at 0 °C for 1h, diluted with dichloromethane and washed with saturated NaHCO_3_ (aq). The organic layer was separated, dried on Na_2_SO_4_ and evaporated. The crude product was purified by flash chromatography on silica using ethyl-acetate:hexane = 1:2 as eluent yielding 15 mg (80%) of product. All spectroscopic data for 4-ONE were in accordance with the previously reported [61, 62] and were as follows for ^1^H NMR (CDCl_3_): δ 0.91 (t, *J* = 6.9 Hz, 3H), 1.26–1.35 (m, 4H), 1.55-1.72 (m, 2H), 2.69 (t, *J* = 7.3 Hz, 2H), 6.77 (dd, *J* = 6.9 and 16.5 Hz, 1H), 6.88 (d, *J* = 16.2 Hz, 1H), 9.78 (d, *J* = 7.2 Hz, 1H).

### Cell culture

N18TG2 cells (Deutsche Sammlung von Mikroorganismen & Zellkultur GmbH (DSMZ), Braunschweig, Germany) were cultivated at 37°C and 5% CO_2_. Cell culture media contained DMEM (21.6 mM glucose) supplemented with 9.6 % fetal bovine serum, 3.85 mM glutamine and 1.92 mM sodium pyruvate (all obtained from Sigma-Aldrich). For experiments cells were cultivated in 4-well Petri dishes (Greiner Bio-One, Germany), coated with poly-D-lysine (Sigma-Aldrich) with 0.5 ml medium per well for 24-72 h before the start of the measurements.

### Microscopy

TMRE was excited at a wavelength of 561 nm with a DPPS laser. Fluorescence was measured with an inverse confocal laser scanning microscope (Leica TCS SP5 II). The microscope was equipped with a heating box for 37°C and 5% CO_2_ supply allowing long-term measurements with living cells. Fluorescence was collected through a 63X water or 40X oil immersion objective in an emission channel of 570 – 690 nm. Z-stacks of cells with a step size of 500 nm (256 × 256 pixels; 400 Hz; 73 frames per z-stack) were recorded every 3 minutes for typically one hour. PI was excited with 514 nm and detected in a 550 – 690 nm channel. NADH levels were imaged with two photon microscopy at an excitation wavelength of 740 nm (emission channel: 400-530 nm) with Chameleon Vision-S laser (Coherent). DAPI fluorescence was imaged after excitation at 700 nm and detected in an emission channel of 400 - 480 nm.

### Measurements of aldehyde dehydrogenase activity

ALDH activity was determined with the AldeRed ALDH detection assay (Merck Millipore) according to the manufacturer's instruction, which is also described in [53]. Briefly, verapamil was dissolved in PBS and added as a 1:100 dilution to the cells (final concentration 24.6 μg/ml). AldeRed 588-A was added in a 1:200 dilution to the verapamil treated cells for 30 min. Cells pre-treated with DEAB solution (dilution 1:100, final concentration 15 μM) were used as a control.

### Cell viability

For cell viability measurements, cells were starved for 2 h 30 min in 25 mM glucose or 3.85 mM glutamine media, and then 16 μM HNE was added for 1 h. Subsequently cells were washed three times. For DAPI staining, cells were fixed in 4% paraformaldehyde and washed again two times. Then cells were stained with 0.6 μM DAPI dissolved in PBS for 5 min and subsequently washed three times for 5 min before analyzing DAPI fluorescence with a fluorescence microscope. For measurements with propidium iodide (PI) cells were stained for 5 min with 1.5 μM PI dissolved in PBS. After washing, PI fluorescence was analyzed with fluorescence microscopy. For the recovery measurements, cells were incubated for three hours in fresh full medium containing 12.5 nM TMRE, before analyzing TMRE fluorescence via fluorescence microscopy.

Caspase-3 activity in N18TG2 neuroblastoma cells was measured using the caspase-3 colorimetric assay kit (Sigma-Aldrich). Cells were exposed to 25 mM glucose or 3.85 mM glutamine containing media for 2 h 30 min, followed by incubation with HNE (1 h). In control experiments cells were maintained in glucose or glutamine media for 3.5 h. After that, cell pellets were centrifuged at 750g / 4°C for 6 min and resuspended in 100 μl of lysis buffer containing 10 mM HEPES, pH 7.4, 1 mM CHAPS, 1 mM DTT. Cell lysates were centrifuged again (12000 g for 1.5 min) and 45 μl of supernatant supplemented with 55 μl assay buffer (containing 2 mM HEPES, pH 7.4, 0.2 mM EDTA, 0.16 mM CHAPS, and 0.5 mM DTT) were filled in 96-well plates per well in duplicates. To one of the wells, the selective caspase 3 inhibitor N-acetyl-Asp-Glu-Val-Asp-CHO (20 μM) was added to record a nonspecific signal. Afterwards, cell lysates were incubated with N-acetyl-Asp-Glu- Val-Asp p-nitroaniline (0.2 mM) for 24 h at 37°C, and the generation of p-nitroaniline (pNA) was measured at 405 nm in a microtiter plate reader. The enzyme activity (μM pNA/d/ml) was calculated as the (OD_sample_ – OD_sample+inhibitor_)/10.5 × 0.045, with 10.5=ε^mM p-nitroaniline^. For determination of HNE-induced caspase 3 activity, the ODs of the corresponding controls without HNE treatment were subtracted from the ODs with HNE treatment.

### Data analysis

Images were analysed with Leica LAS AF Lite software. Fluorescence levels in cells were corrected for the background. All data are presented as mean ± SEM. p-values were determined with a two sample t-test with unequal variances and classified with * p < 0.05, ** p < 0.01, *** p < 0.001.

## Abbreviations

ALDH: aldehyde dehydrogenase
CCCP: Carbonyl cyanide m-chlorophenyl hydrazone
DEAB: diethylaminobenzaldehyde
DMEM: Dulbecco's Modified Eagle Medium
LCFA: long chain fatty acids
MMP: mitochondrial membrane potential
HNA: 4-hydroxy-2-nonenoic acid
HNE: 4-hydroxy-2-nonenal (HNE)
ONE: 4-oxo-2-nonenal
PI: propidium iodide
PUFAs: polyunsaturated fatty acids
TMRE: tetramethyl rhodamine ethyl ester
TTFA: thenoyltrifluoroacetone
